# Investigation of the Vibrational Characteristics of 6-Isocyano-1-Methyl-1H-Indole: Utilizing the Isonitrile Group as an Infrared Probe

**DOI:** 10.3390/molecules28196939

**Published:** 2023-10-05

**Authors:** Min You, Zilin Gao, Liang Zhou, Changyuan Guo, Qiang Guo

**Affiliations:** 1 School of Computer Science and Engineering, Chongqing Three Gorges University, Wanzhou, Chongqing 404100, China; 2 Department of Physics and Applied Optics Beijing Area Major Laboratory, Center for Advanced Quantum Studies, Beijing Normal University, Beijing 100875, China; 201621140066@mail.bnu.edu.cn; 3 Key Laboratory of Intelligent Air-Ground Cooperative Control for Universities in Chongqing, College of Automation, Chongqing University of Posts and Telecommunications, Chongqing 400065, China; 4 Shanxi Province Key Laboratory of Higee-Oriented Chemical Engineering, School of Chemistry and Chemical Engineering, North University of China, Taiyuan 030051, China

**Keywords:** indole derivative, infrared probe, isonitrile group, vibrational characteristics, infrared spectroscopy, theoretical calculations

## Abstract

Indole derivatives have garnered considerable attention in the realm of biochemistry due to their multifaceted properties. In this study, we undertake a systematic investigation of the vibrational characteristics of a model indole derivative, 6-isocyano-1-methyl-1H-indole (6ICMI), by employing a combination of FTIR, IR pump-probe spectroscopy, and theoretical calculations. Our findings demonstrate a strong dependence of the isonitrile stretching frequency of 6ICMI on the polarizability of protic solvents and the density of hydrogen-bond donor groups in the solvent when the isonitrile group is bonded to aromatic groups. Both experimental and theoretical analyses unveil a significant correlation between the isonitrile stretch vibration of 6ICMI and the solvent acceptor number of alcohols. Furthermore, the polarization-controlled infrared pump-probe conducted on 6ICMI in dimethyl sulfoxide provides additional support for the potential use of the isonitrile stretching mode of 6ICMI as an effective infrared probe for local environments.

## 1. Introduction

Indole derivatives have attracted considerable attention in the field of biochemistry due to their diverse properties. These derivatives exhibit a broad range of beneficial effects, including anti-cancer [1,2], anti-bacterial [3,4], anti-HIV [5,6], anti-oxidant [7], anti-diabetic [8,9], anti-inflammatory [10,11,12,13], and anti-fungal activities [14,15]. One prominent example of an indole derivative is Tryptophan (Trp), which has proven valuable in exploring various protein functions by incorporating different functional groups onto the indole ring. Specifically, cyano-tryptophans have been used as fluorescent [16,17,18,19] and infrared (IR) probes [20,21,22], enabling the investigation of local environments surrounding proteins. Among the cyano-tryptophans, 5-cyanoindole has been employed as an effective IR probe due to its linear correlation between the nitrile stretching vibration and solvent parameters [20]. Similarly, 4-cyanoindole has been utilized to assess the hydration status within a local environment, exploiting its Fermi resonance properties [22]. Furthermore, the incorporation of 5-cyanoindole-Trp has allowed for the examination of hydrogen-bonding near the Trp gate of the influenza A M2 proton channel [21]. However, the use of cyano-tryptophans probes in nonlinear IR spectroscopy encounters certain challenges that limit their feasibility in measurements. These challenges stem from the relatively weak signal generated by the limited transition dipole strength of the cyano-tryptophans probe, as well as its low concentration within biological samples. Consequently, extensive research efforts have been dedicated to the development of new IR probes, such as ones incorporating ester or isonitrile groups onto the indole ring [23,24,25], to overcome these limitations.

**Figure 1:**
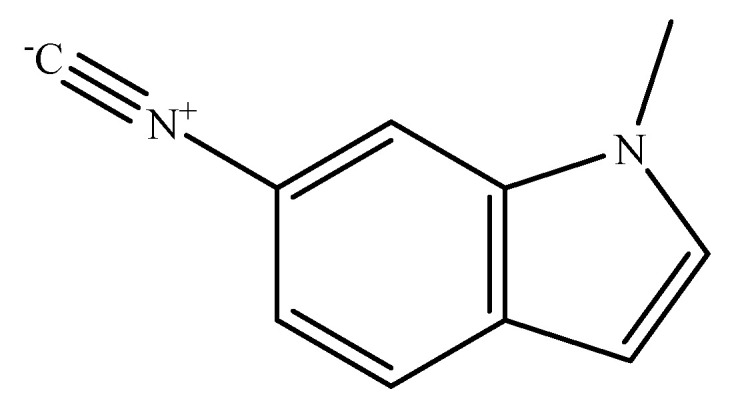
Structure of the 6-isocyano-1-methyl-1H-indole (6ICMI).

This study aimed to investigate the vibrational properties of new indole derivative, 6-isocyano-1-methyl-1H-indole (6ICMI), depicted in Figure 1. By analyzing its behavior in various solvents, we can determine its suitability as an excellent IR probe. Initially, Fourier Transform Infrared (FTIR) spectroscopy was employed to examine the behavior of 6ICMI in different solvents. The influence of solvents on the vibrational properties of the isonitrile group was assessed using the well-established Kamlet–Taft empirical parameters [26,27]. In addition, other solvent parameters including the solvent acceptor number (AN), the solvent donor number (DN), and the Kirkwood–Bauer–Magat (KBM) solvation parameter *f* (*f* = (ε − 1)/(2ε + 1)) were also considered [28,29]. AN is a quantitative empirical parameter for the electrophilic properties of solvents [30], while DN could describe the nucleophilic behavior of solvents [31]. All the parameters were presented in detail in Table 1. Furthermore, to gain deeper insights into the sensitivity of the isonitrile stretching vibration frequency to the solvents, theoretical calculations were conducted for 6ICMI immersed in various solvent systems. Finally, we employed a model solvent dimethyl sulfoxide (DMSO) to investigate the dynamics properties of 6ICMI in solvents using polarization-controlled IR pump-probe measurements.

## 2. Results and Discussion

### 2.1. FTIR Spectroscopy

A study was conducted to examine the isonitrile stretching vibration of 6ICMI in a selected pure solvent at room temperature (Figure S1). The obtained spectra showed the presence of at least two distinct peaks. One peak was consistently observed around 2102 cm^−1^, while the other peak exhibited unique spectral features depending on the solvents employed. To analyze the isonitrile vibrational spectral characteristics, each peak was fitted using a pseudo-Voigt function profile, which yielded a satisfactory fit to the experimental data obtained from all solvents studied (Table 1). The isonitrile stretching frequency of 6ICMI was previously determined to be approximately 2120 cm^−1^ based on reference work [24,32], and it was found to be significantly influenced by the choice of solvents. However, the peak observed at around 2102 cm^−1^ appeared to be independent of the solvents used. The precise assignment of this peak remains uncertain, but it is hypothesized that it may arise from the presence of Fermi resonance in 6ICMI. Previous studies have utilized Fermi resonance to elucidate the hydrogen-bonding characteristics of different probes [22]. Consequently, it is imperative to conduct further research to explore the nature of this peak and expand our understanding in future investigations. In this paper, our focus is solely on the spectral characteristics of the isonitrile vibration of 6ICMI above 2110 cm^−1^ in different solvents. Therefore, any discussion or analysis in the subsequent sections disregards the lower left peaks. To facilitate the comparison, the Supplementary Materials contain extensive spectral data related to the isonitrile vibration of two compounds: 5-isocyano-1-methyl-1H-indole (5ICMI) and 2-naphthyl isocyanide (2NI). These dates are presented in Table S1 and Table S2, respectively.

**Figure 2:**
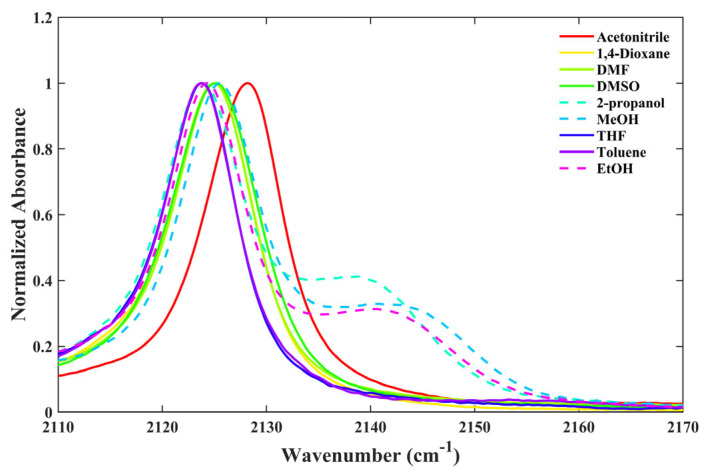
The isonitrile stretching vibration of 6ICMI in selective solvents.

Table 1 presents the results obtained from the dissolution of 6ICMI in various solvents. As shown in Figure 2, a single peak corresponding to the isonitrile stretch was observed in aprotic solvents such as acetonitrile, 1,4-dioxane, DMF, DMSO, THF, and toluene. However, when 6ICMI was dissolved in alcohols, the appearance of two distinct stretching peaks suggested the coexistence of non-hydrogen-bonded and hydrogen-bonded isonitrile groups within the alcohol solvents (represented by the dashed line in Figure 2). Firstly, we discuss the isonitrile stretch vibration of the former, which was observed at approximately 2123 cm^−1^, denoted as *w*_0(NC)_. Comparing the *w*_0(NC)_ value of 6ICMI with that in *n*-octanol, a shift of approximately 6 cm^−1^ was observed in acetonitrile (see Table 1). This shift in the stretching frequency can be attributed to the impact of the solvent’s polarizability, which induced a local electric field affecting the *w*_0(NC)_ value and causing the frequency shift. When we transitioned from toluene to acetonitrile, about a 4 cm^−1^ shift of *w*_0(NC)_ was observed in the aprotic solvents. This observed shift suggests a decrease in the π* or ε parameters, as other factors remained fairly constant between these two solvents. Hence, this observed shift can be associated with changes in the π* or ε parameters.

**Figure 3:**
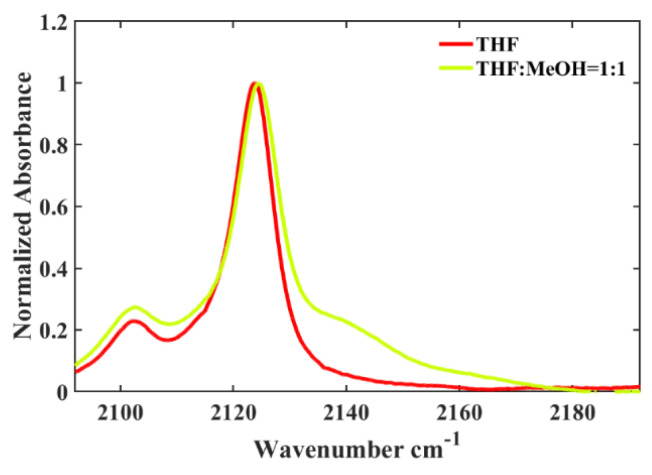
Comparison of the *w*_0(NC)_ of 6ICMI in pure THF and in a binary solvent with a volume ratio of THF to methanol of 1:1 (*V*_THF_:*V*_MeOH_ = 1:1).

Furthermore, it has been demonstrated that the hydrogen-bonding capability of the solvent exerts a substantial impact on the isonitrile stretching vibration. For instance, similar to the behavior observed in 5ICMI, a solvent switch from THF to methanol (MeOH), characterized by comparable π* and β values but differing α values, caused an about 2 cm^−1^ shift in the *w*_0(NC)_ of 6ICMI. Additionally, in protic solvents, the bandwidth of the isonitrile stretching mode was slightly broader compared to that in aprotic solvents (Figure 3). This broadening can be attributed to the H-bonding interactions between the samples and solvents, consistent with the previous findings [22,24]. In aprotic solvents, the frequency and bandwidth of the isonitrile stretch vibration were comparable, despite significant differences in the corresponding polarizabilities of the solvents. These findings collectively suggest that the *w*_0(NC)_ of 6ICMI is sensitive to the hydrogen-bonding donor (HBD) ability of the solvents.

**Figure 4:**
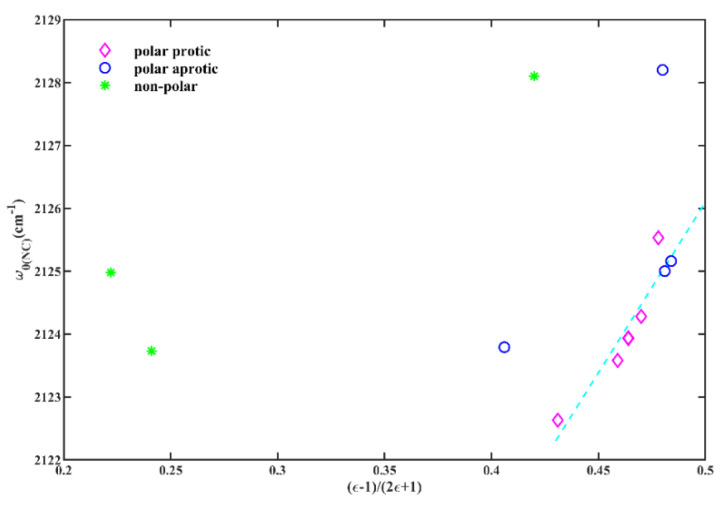
The *w*_0(NC)_ of 6ICM versus the solvent parameter (ε − 1)/(2ε + 1).

These comparisons offer compelling evidence regarding the influence of solvent polarizability and hydrogen-bonding capacity on the sensitivity of the isonitrile group. However, a comprehensive quantitative analysis of the FTIR spectra is necessary to better comprehend the specific contributions of these interactions. While Figure S2 shows that there is not a straightforward linear relationship between the *w*_0(NC)_ of 6ICMI and solvent parameters across all examined solvents, Figure 4 indicates that the results suggest a discernible dependence of *w*_0(NC)_ on the KBM solvation parameters, except in non-polar solvents. It is important to note that the KBM model used in this study is a simplified theoretical approach [29] which may not fully capture the intricacies of real solutes and solvents. Nevertheless, it suggests a correlation between the symmetric stretching of isonitriles and the polarity and proticity of solvents. Protic solvents, such as alcohols, have the capability to form hydrogen bonds, which can influence the isonitrile band. Additionally, the polarizability of the solvent can also have an impact on the isonitrile band.

**Figure 5:**
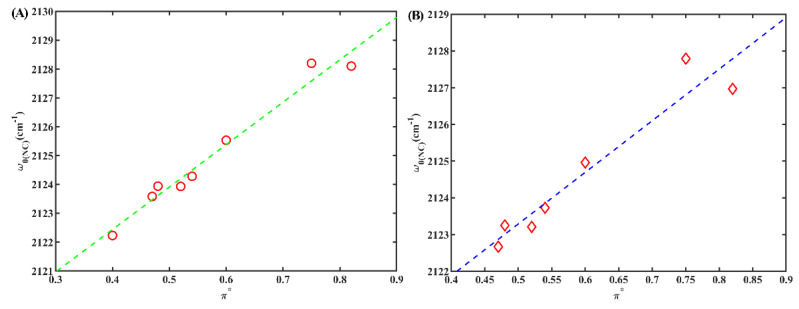
(**A**) The *w*_0(NC)_ of 6IMCI versus solvent parameter π*; only frequencies obtained in protic solvents (*α* ≠ 0) were used. (**B**) The *w*_0(NC)_ of 2NI versus solvent parameter π*; only frequencies obtained in protic solvents (α ≠ 0) were used.

To differentiate between the solvatochromic effects attributed to hydrogen bonding and polarizability, we initially directed our attention towards aprotic solvents with a Kamlet–Taft parameter (α) value of zero (as indicated in Table 1). We investigated the correlations between *w*_0(NC)_ and those parameters in these aprotic solvents. However, no linear relationships were observed between *w*_0(NC)_ and any the solvent parameters (Figure S3). These findings align with previous studies [24,32]. Interestingly, a strong linear relationship was observed between *w*_0(NC)_ and polarizability (π*) when α was not zero (Figure 5A) (R^2^ = 0.99, where R-squared signifies the coefficient of determination for linear regression). This suggests that *w*_0(NC)_ can serve as a sensitive indicator of the polarizability of protic solvents when the isonitrile group is bonded to an indole ring. Notably, the slope of the linear relationship for 6ICMI was slightly larger than that of 5ICMI (14.7 vs. 13.3). This observation suggests that the *w*_0(NC)_ of 6ICMI displayed a higher degree of responsiveness to variations in the dielectric constant of solvents when compared to that of 5ICMI. As a result, 6ICMI possesses the potential to serve as a more sensitive infrared probe.

Subsequently, we conducted further investigations to clarify the relationship between *w*_0(NC)_ and the polarizability of protic solvents in cases where the isonitrile group is not bonded to an indole ring. Remarkably, our findings revealed a robust linear relationship (R^2^ = 0.98) even when the isonitrile group was attached to a naphthalene ring (Figure 5B). However, a previous report indicated that no significant linear relationship was observed when the isonitrile group was bonded to an aliphatic carbon [32]. These results suggest that the presence of bulky volume groups, such as an indole ring or a naphthalene ring, confers stability to the isonitrile group, thereby mitigating interference from neighboring groups concerning the polarizability of protic solvents. Therefore, it can be reasonably concluded that the isonitrile group can effectively function as a probe to evaluate the polarizability of protic solvents when it is attached to bulky groups such as an indole ring or a naphthalene ring.

**Figure 6:**
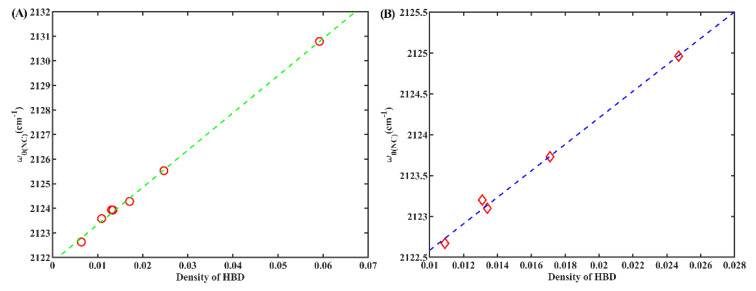
(**A**) The *w*_0(NC)_ of 6ICMI versus the density of HBD groups in solvents. (**B**) The *w*_0(NC)_ of 2NI versus the density of HBD groups in solvents.

Furthermore, we also discovered a pronounced linear dependency (R^2^ = 0.95) between *w*_0(NC)_ and the density of HBD groups in the solvent (Figure 6A). The density of HBD groups can be determined using the formula ρ*n*/*M*, where ρ denotes the density of the solvent, *M* corresponds to the molar mass of each solvent, and *n* represents the count of HBD groups in each solvent molecule. Similar findings have been reported for other IR probes [24,33,34]. These observations indicate that the *w*_0(NC)_ of 6ICMI exhibits sensitivity to the local density of HBD groups, as well. Comparing the sensitivity between 6ICMI and 5ICMI, it shows that the slope for 6ICMI in this linear relationship exceeded that of 5ICMI (151.5 vs. 138.0). This indicates that 6ICMI is more suitable as an IR probe than 5ICMI. Interesting, a similar linear relationship was observed for 2NI, as depicted in Figure 6B. Hence, it can be inferred that the isonitrile group is an excellent choice as a highly sensitive infrared probe to detect the density of HBD groups within a local environment, particularly when it is attached to aromatic groups.

**Figure 7:**
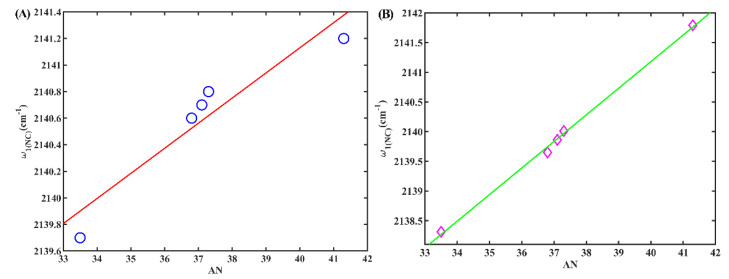
(**A**) The *w*_1(NC)_ of 6ICMI versus the solvent parameter AN. (**B**) The *w*_1(NC)_ of 5ICMI versus the solvent parameter AN.

Finally, we examined the occurrence of a secondary peak at approximately 2140 cm^−1^ in alcohols, which we qualitatively attribute to the stretching mode of the hydrogen-bonded isonitrile group (*w*_1(NC)_). This attribution is supported by our previous research [24] and our following calculations. To understand the relationship between *w*_1(NC)_ and the corresponding solvent Kamlet–Taft parameters, we conducted an investigation. It was found that there was no linear correlation between these parameters, as depicted in Figure S4. Similarly, we did not observe a linear correlation between *w*_1(NC)_ and the density of the HBD, either, as depicted in Figure S5. However, a noteworthy outcome arose during our investigation. We identified a strong linear correlation between *w*_1(NC)_ and AN (R^2^ = 0.97), as illustrated in Figure 7A. AN is an empirical parameter developed by Gutmann based on the P-NMR chemical shifts of triethylphosphane oxide in different solvents [28] and serves as an indicator of solvent electrophilicity. This linear correlation between *w*_1(NC)_ and AN was also evident for 5ICMI, as demonstrated in Figure 7B. Based on these findings, it can be inferred that the isonitrile group can serve as a valuable probe for evaluating the electrophilicity of protic solvents, despite the weaker intensity of the shoulder peak compared to the main peak in these solvents.

### 2.2. Quantum Chemical Calculation

In order to gain insight into the underlying molecular factors contributing to the solvent sensitivity observed in the isonitrile stretch frequency, we conducted theoretical calculations on 6ICMI across various solvent environments. First, 6ICMI was optimized in the gas phase and in the conductor-like dielectric continuum model (CPCM) implicit solvation model. Solvation energies, frequencies, and the isonitrile bond length are shown in Table 2. Predictions for gas-phase 6ICMI are shown; no experimental spectra documenting the gas-phase characteristics of 6ICMI have been reported in the available references and associated experiments, to the best of our knowledge.

**Figure 8:**
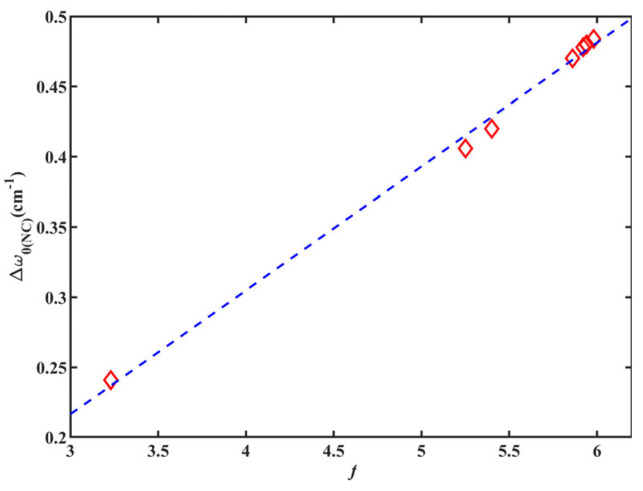
Calculated Δ*w*_0(NC)_ of 6ICMI versus solvent parameter of KBM (*f*).

According to the information presented in Table 2, the calculated value of *w*_0(NC)_ for 6ICMI closely align with the experimental result. Additionally, the CPCM model exhibited remarkable agreement with the KBM relationship in accurately predicting frequency shifts, as depicted in Figure 8. The dipole moment of 6ICMI increased proportionally with the solvent’s dielectric constant, as supported by a strong correlation (R^2^ = 0.995). Consequently, the *w*_0(NC)_ of 6ICMI experienced a commensurate blueshift from its gas-phase value. A sharp rise in frequency was observed as the dielectric constant increased from unity in vacuum (*w*_0(NC)_= 2117.3 cm^−1^) to 3.2 in toluene (*w*_0(NC)_ = 2120.5 cm^−1^) (Table 2). However, selecting solvents with higher dielectric constants than toluene had a minimal impact on the frequency, since the scaling factor asymptotically approached the value of the half. The “plateau” region was observed from THF with ε of 7.5, where the dichloromethane (DCM), ethanol, methanol, acetonitrile, and DMSO yield calculated frequencies within 1 cm^−1^ of each other (Table 2). Experimentally, no such plateau region was present in the observed frequencies (Table 1), indicating that the alterations in the distribution of electron density contributing to the isonitrile vibration shift cannot be solely attributed to electrostatics. Thus, local interactions between the first solvation shell and the solute, particularly for protic solvents, must be considered.

**Figure 9:**
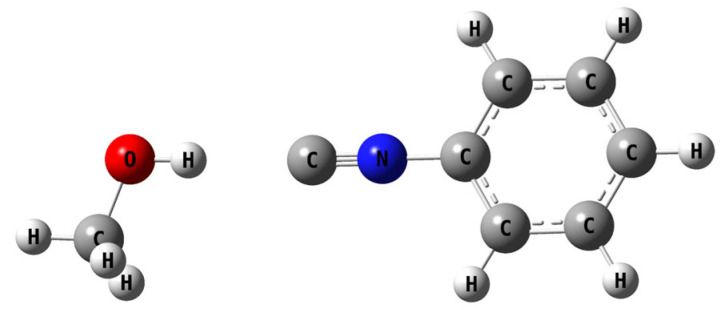
A model for the lowest energy structures in which the solvent is interacting with the isonitrile.

Considering the minimal discrepancies observed in the *w*₀_(NC)_ calculations of 6ICMI and isocyanobenzene (ICB) performed under identical conditions, we selected ICB in explicit solvents as simplified models to investigating the hydrogen bonding interactions between solute and solvent molecules. Multiple conformations of the ICB–solvent complexes were subsequently optimized, with an emphasis on protic solvents interacting with the isonitrile group, as shown in Figure 9. These complexes were further optimized in an implicit solvent environment. The B3LYP/6-31+G(d,p) level of theory was employed to calculate zero-point corrected binding energies (or solvation energies in the case of implicit solvent) and frequencies. The corresponding results are listed in Table 3.

**Figure 10:**
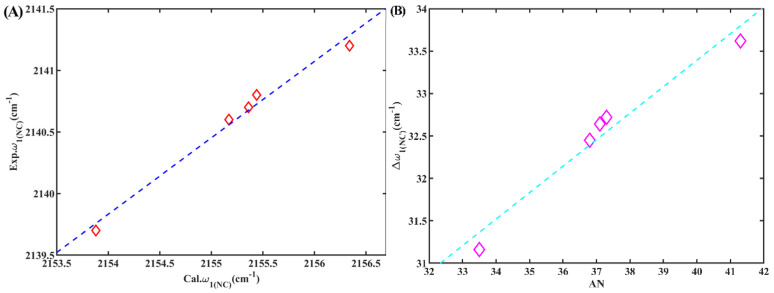
(**A**) The calculated *w*_1(NC)_ of ICB versus the experimental *w*_1(NC)_ of 6ICMI in the corresponding solvent. (**B**) The calculated Δ*w*_1(NC)_ of ICB versus solvent parameter AN.

Although the calculated frequencies of these complexes exhibited higher values than their corresponding experimental counterparts, the observed trend in the changes of *w*_1(NC)_ remained consistent (Figure 10A). The interaction between solvents and the isonitrile group led to a blueshift in the stretching frequency compared to the vacuum, accompanied by a proportional reduction in the length of the isonitrile bond. Interestingly, a strong linear correlation (R^2^ = 0.96) was observed between *w*_1(NC)_ of these complexes and AN, which closely agreed with the relationship observed in experiments (Figure 10B). Consequently, this suggests that the isonitrile group bonded to the aromatic group could indeed serve as an effective IR probe for detecting the local environment.

### 2.3. Polarization-Controlled IR Pump-Probe Spectroscopy

**Figure 11:**
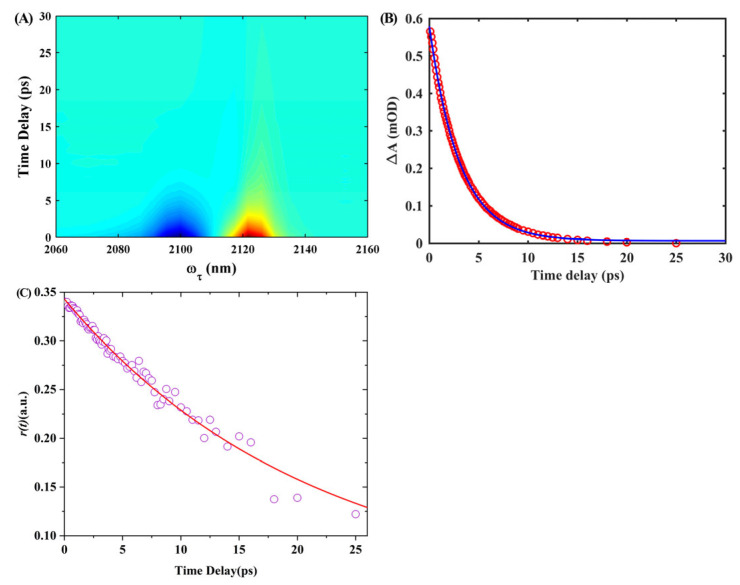
(**A**) Time- and frequency-resolved isotropic IR pump-probe signals for 6ICMI in DMSO. (**B**) Transient absorption spectra as a function of probe delay time and frequency for 6ICMI in DMSO. The signal can be fitted to a single exponential function (blue line). (**C**) Anisotropy decays of 6ICMI in DMSO. The signal can be fitted to a single exponential function (red line).

To investigate the lifetimes of vibrational and orientational relaxation of the isonitrile stretch mode in the 6ICMI when dissolved in solvents, we conducted IR pump-probe measurements with polarization control. We specifically chose DMSO as a representative solvent. Figure 11A shows the corresponding frequency-resolved IR pump-probe signals of 6ICMI dissolved in DMSO. These signals consist of positive and negative components that arise from different transition pathways. The positive peak corresponds to the ground-state bleach (GSB) and stimulated emission (SE), which involve transitions from the ground state to the first excited state and back. On the other hand, the negative peak arises from excited-state absorption (ESA), which involves a transition from the first excited state to the second excited state. Therefore, the negative peak centered at 2102 cm^−1^ was attributed to GSB and SE, while the positive peak centered at 2125 cm^−1^ was associated with ESA. The difference in frequency between the GSB and ESA peaks provided information about the vibrational anharmonicity and line-broadening effects. As time progressed, the energy relaxation process caused the signal to decay and eventually reach zero at longer time delays.

In order to determine the vibrational lifetime of *w*_0(NC)_ for 6ICMI in DMSO, we performed an analysis of the time profiles by observing the integrated peak areas of the positive peaks (Figure 11B). These profiles were then fitted using a single exponential function. The results obtained revealed a time constant of 6.26 ± 0.13 ps, which corresponded to the vibrational lifetime of 6ICMI in DMSO. It is worth noting that this vibrational lifetime was slightly longer compared to that of 5-isocyano-1H-indole (5ICI) (6.3 vs. 5.4 ps) [24]. It was significantly longer than the lifetimes observed for other vibrational modes, such as the azido stretching mode and the nitrile group stretching mode [32].

Furthermore, we also determined the orientational relaxation time constant for the isonitrile stretch of 6ICMI in DMSO (Figure 11C). The decay of anisotropy was fitted using a single exponential function, yielding a time constant of 21.0 ± 1.1 ps, which was still longer than the orientational relaxation time of 5ICI (21.0 vs. 15.6 ps) [24]. It exceeded the relaxation time displayed by the isonitrile stretch of isonitrile-derivatized alanine in DMF by a significant margin, as well [32]. These findings suggest that the relatively longer orientational relaxation time associated with the isonitrile stretch in 6ICMI provides an opportunity to gain more efficient insights into local intermolecular interactions, similar to those observed with 5ICI. Overall, these results indicate that 6ICMI functions as a sensitive infrared probe, enabling the investigation of local environments with greater effectiveness.

## 3. Materials and Methods

### 3.1. Materials and Sample Preparation

The compound 6ICMI was obtained from Sigma-Aldrich. Various solvents were acquired from either Sigma-Aldrich or J&K Scientific and utilized without further purification. Prior to use, the 6ICMI compound was dissolved in the appropriate solvent to achieve a final concentration of approximately 50 mM. Subsequently, the prepared sample solutions were placed between two CaF_2_ windows, with a spacer of either 100 or 200 μm, for conducting spectroscopic measurements in this study. All the sample solutions were freshly prepared before each experimental procedure.

### 3.2. Spectroscopic Measurements

All Fourier Transform Infrared (FTIR) spectra were acquired using a Bruker VERTEX 70 spectrometer with a frequency resolution of 0.5 cm^−1^ at 22 °C. Detailed information on the experimental configuration for ultrafast IR spectroscopy can be found elsewhere [35]. In brief, the setup involved the independent operation and synchronization of a picosecond (ps) amplifier and a femtosecond (fs) amplifier using the same seed pulse generated from a Ti-sapphire oscillator. The ps amplifier drove an Optical Parametric Amplifier (OPA) to generate approximately 1 ps Mid-IR pulses, with a bandwidth of around 18 cm^−1^, at a repetition rate of 1 kHz. The fs amplifier, on the other hand, powered another OPA to produce approximately 140 fs Mid-IR pulses, with a bandwidth of roughly 200 cm^−1^ and also at a repetition rate of 1 kHz.

For the polarization-selective IR pump-probe experiments, the ps IR pulse acted as the pump beam, while the fs IR pulse served as the probe beam and underwent frequency resolution using a spectrograph. To selectively measure the parallel or perpendicular polarized signal relative to the pump beam, two polarizers were introduced into the probe beam path. Vibrational lifetimes were determined by analyzing the rotation-free signal using the equation S_life_ = S|| + 2S⊥, where S|| and S⊥ represent the parallel and perpendicular signals, respectively. Rotational relaxation times were derived from the anisotropy that varied with the waiting time, calculated as *r*(*t*) = (S|| − S⊥)/(S|| + 2S⊥).

### 3.3. Computational

Electronic structure calculations were conducted employing Gaussian 09, revision A.02 [36], with an optimization of structures and a calculation of vibrational frequencies performed at the B3LYP/6-31+G(d,p) level. In the optimized geometry of the molecules, no imaginary frequency modes were observed, indicating the presence of a true energy minimum on the potential energy surface. Single point energy calculations were subsequently carried out on the optimized structures. In order to account for the solvation effects, the conformational analysis of 6ICMI was investigated both in a vacuum and employing the CPCM solvation models [37]. Computational investigations encompassed six solvents: toluene, DCM, DMF, acetonitrile, MeOH, ethanol, and THF. In order to assess the influence of hydrogen bonding, explicit solvent molecules including MeOH, ethanol, 1-propanol, 2-propanol, and *n*-butanol were included. These explicit solvent systems were studied in various configurations wherein the solvent molecules engaged in hydrogen bonding interactions with the isonitrile group of 6ICMI. Moreover, these explicit solvent systems were investigated both in combination with implicit solvents and in a vacuum.

## 4. Conclusions

In summary, this study aimed to investigate the vibrational characteristics of 6ICMI utilizing FTIR, IR pump-probe spectroscopy, and theoretical calculations. The obtained results provided insights into the relationship between the frequency of the isonitrile group and the solvent parameters of KBM. Notably, a strong linear correlation was observed between the center frequency of the isonitrile stretching mode and the polarizability of polar solvents. Both experimental and theoretical analyses indicated that the isonitrile stretch vibration of 6ICMI significantly depended on the solvent acceptor number of alcohols, thus suggesting that the isonitrile frequency could serve as an indicator of solvent polarizability and electrophilicity.

Additionally, this research established a notable relationship between the isonitrile stretching frequency of 6ICMI and the density of hydrogen-bond donor groups in solvents, highlighting its superiority over 5ICMI. Furthermore, the investigation of the dynamic properties of 6ICMI in DMSO revealed a surprising result: the lifetime of the isonitrile stretching vibration of 6ICMI, when dissolved in DMSO, exceeded that of isonitrile-derivatized alanine in DMF and 5ICI in DMSO. These findings provide conclusive evidence to support the potential application of 6ICMI as an excellent infrared probe for evaluating local environments.

## Figures and Tables

**Table 1:** Frequency parameters and solvent parameters for 6ICMI in different solvents. The center frequency (*w*_0(NC)_, cm^−1^), and the shoulder frequency (*w*_1(NC)_) of the isonitrile stretching band of 6ICMI in various solvents and each solvent with its Kamlet–Taft parameters, π* (polarizability), β (hydrogen bond acceptor), α (hydrogen bond donor), and ε (dielectric constant) are listed in the table. Other solvent parameters like AN (acceptor number), DN (donor number), and *f* (KBM solvation parameter) are listed in the table as well.

Solvent	*w*_0(NC)_	*w*_1(NC)_ ^a^	π*	β	α	ε	AN ^b^	DN	*f*
*n*-octanol	2122.6	2139.4	0.4	0.81	0.77	10.3		32	0.431
acetonitrile	2128.2		0.75	0.31	0.19	37.5	18.9	14.1	0.480
1,4-dioxane	2124.9		0.55	0.37	0	2.2	10.3	14.3	0.222
N,N-Dimethylformamide (DMF)	2125.0		0.88	0.69	0	38.2	16	26.6	0.481
dimethyl sulfoxide (DMSO)	2125.2		1	0.76	0	47.2	19.3	29.8	0.484
2-propanol	2123.9	2139.7	0.48	0.95	0.76	20.2	33.5	36	0.464
methanol (MeOH)	2125.5	2141.5	0.6	0.62	0.93	33	41.3	30	0.478
tetrahydrofuran (THF)	2123.8		0.58	0.55	0	7.5	8	20	0.406
*n*-butanol	2123.6	2140.6	0.47	0.88	0.79	17.8	36.8	29	0.459
toluene	2123.7		0.54	0.11	0	2.4		0.1	0.241
*n*-propanol	2123.9	2140.8	0.52	0.9	0.84	20.1	37.3	19.8	0.464
ethanol(EtOH)	2124.3	2140.7	0.54	0.77	0.83	24.5	37.1	32	0.470
dichloromethane (DCM)	2128.1		0.82	0.1	0.13	8.9	20.4	1	0.420

^a^ Blanks in this column indicate the absence of distinct shoulder peaks observed for 6ICMI in the corresponding solvent. ^b^ Blanks in this column signify the lack of available data to the best of our knowledge.

**Table 2:** The 6ICMI in implicit solvent. Properties of the isonitrile group for 6ICMI as predicted by CPCM implicit solvent with no solvent ligand are listed in the table, such as solvation energies *E*_solvation_ (kcal/mol), dipole moment *m* (Debye), *w*_0(NC)_ (cm^−1^), N≡C bond length L_(NC)_ (Å), and *f*. Changes are reported with gas phase 6ICM as the reference state. The solvation energies are zero-point corrected.

Solvent	*E*_solvation_	*m*	Δ*m*	Cacl.*w*_0(NC)_	Δ*w*_0(NC)_	*L*_(NC)_	Δ*L*_(NC)_	*f*
Vacuum		5.8551		2121.26		1.1784		
Toluene	−14.0895	6.7521	0.8970	2124.49	3.24	1.1769	−0.0014	0.241
THF	−22.2480	7.3319	1.4768	2126.51	5.26	1.1761	−0.0022	0.406
DCM	−22.9351	7.3831	1.5280	2126.67	5.41	1.1761	−0.0023	0.420
Ethanol	−25.1526	7.5509	1.6958	2127.13	5.88	1.1759	−0.0025	0.470
MeOH	−25.4537	7.5740	1.7189	2127.19	5.93	1.1758	−0.0025	0.478
Acetonitrile	−25.5372	7.5805	1.7254	2127.21	5.95	1.1758	−0.0025	0.480
DMSO	−25.7478	7.5967	1.7416	2127.25	5.99	1.1758	−0.0026	0.484

**Table 3:** Properties of the isonitrile group of ICB interacting with explicit solvent ligand in implicit solvent are listed in the table, such as solvation energies *E*_solvation_ (kcal/mol), *w*_1(NC)_ (cm^−1^), N≡C bond length *L*_(NC)_ (Å), AN, and *f.* Solvation energies calculated as (*E*_complex,CPCM_ − *E*_solute,vacuum_ − *E*_solvent,CPCM_).

Explicit Solvent	*E*_solvation_	Calc. *w*_1(NC)_	Exp. *w*_1(NC)_	Δ*w*_1(NC)_	*L*_(NC)_	Δ*L*_(NC)_	AN	*f*
Vacuum		2122.66			1.17836			
2-propanol	−28.3289	2153.88	2139.70	31.22	1.17318	−0.00518	33.5	0.464
*n*-butanol	−29.7850	2155.17	2140.60	32.51	1.17300	−0.00536	36.8	0.459
Ethanol	−29.9885	2155.36	2140.70	32.70	1.17297	−0.00539	37.1	0.470
1-propanol	−29.9892	2155.44	2140.80	32.78	1.17297	−0.00539	37.3	0.464
MeOH	−30.5860	2156.34	2141.20	33.68	1.17283	−0.00553	41.3	0.478

## Data Availability

The data presented in this study are available on request from the corresponding author.

## Supplementary Materials

The following supporting information can be downloaded at: https://www.mdpi.com/article/10.3390/molecules28196939/s1, Figure S1: The isonitrile stretching vibration of 6ICMI in selected solvents; Figure S2. No linear relationships were observed between *w*_0(NC)_ of 6ICMI and any solvent parameters in all studied solvents; Figure S3. No linear relationships were observed between *w*_0(NC)_ of 6ICMI and any solvent parameters in aprotic solvents; Figure S4. No linear relationships were observed between *w*_1(NC)_ of 6ICMI and any other Kamlet–Taft parameters in studied solvents; Figure S5. No linear relationships were observed between *w*_1(NC)_ of 6ICMI and the density of hydrogen-bond donor groups in solvents; Table S1. Frequency parameters and solvent parameters for 5ICMI in different solvents. The center frequency (*w*_0(NC)_, cm^−1^) and the shoulder frequency (*w*_1(NC)_) of the isonitrile stretching band of 5ICMI in various solvents and each solvent with its Kamlet–Taft parameters, π*(polarizability), β (hydrogen bond acceptor), α (hydrogen bond donor), and ε (dielectric constant) are listed in the table. KBM solvent parameter *f* is listed in the table as well; Table S2. Frequency parameters and solvent parameters for 2NI in different solvents. The center frequency (*w*_0(NC)_, cm^−1^) of the isonitrile stretching band of 2NI in various solvents and each solvent with its Kamlet–Taft parameters, π*(polarizability), β (hydrogen bond acceptor), α (hydrogen bond donor), and ε (dielectric constant) are listed in the table.
