# Supplementary material for: Investigation of the Vibrational Characteristics of 6-Isocyano-1-Methyl-1H-Indole: Utilizing the Isonitrile Group as an Infrared Probe
Source: Molecules. 2023 Oct 5;28(19):6939. doi: 10.3390/molecules28196939 (PMC10574170; doi:10.3390/molecules28196939)
Supplement: Supplementary file 1 [file molecules-28-06939-s001.zip › molecules-2639091-supplementary.pdf]

Supplementary Material for:

Investigation of the Vibrational Characteristics of 6-Isocyano-1-Methyl-1H-Indole: Utilizing the Isonitrile Group as an Infrared Probe

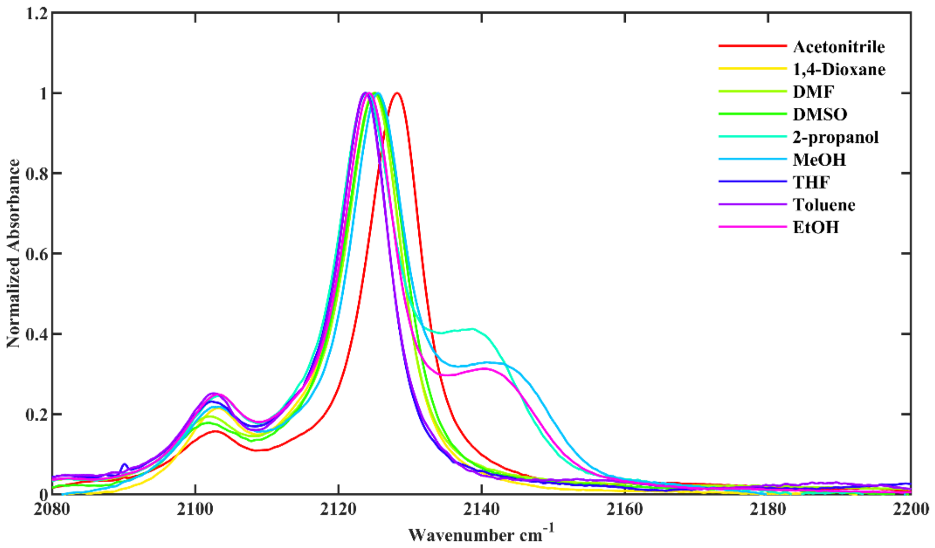

Figure S1. The isonitrile stretching vibration of 6ICMI in selected solvents.

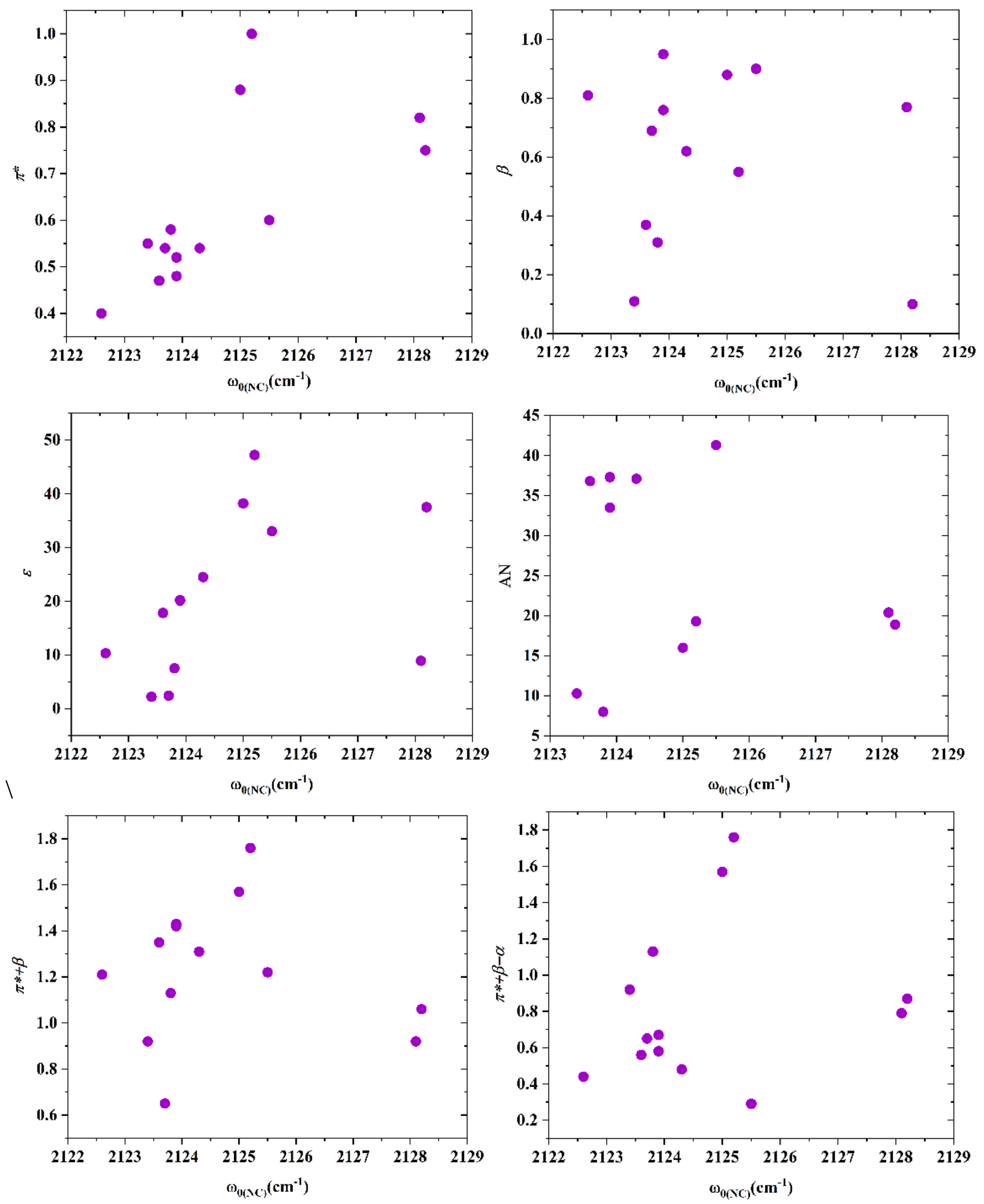

Figure S2. No linear relationships were observed between $w_{0(NC)}$ of 6ICMI and solvent parameters in all studied solvents.

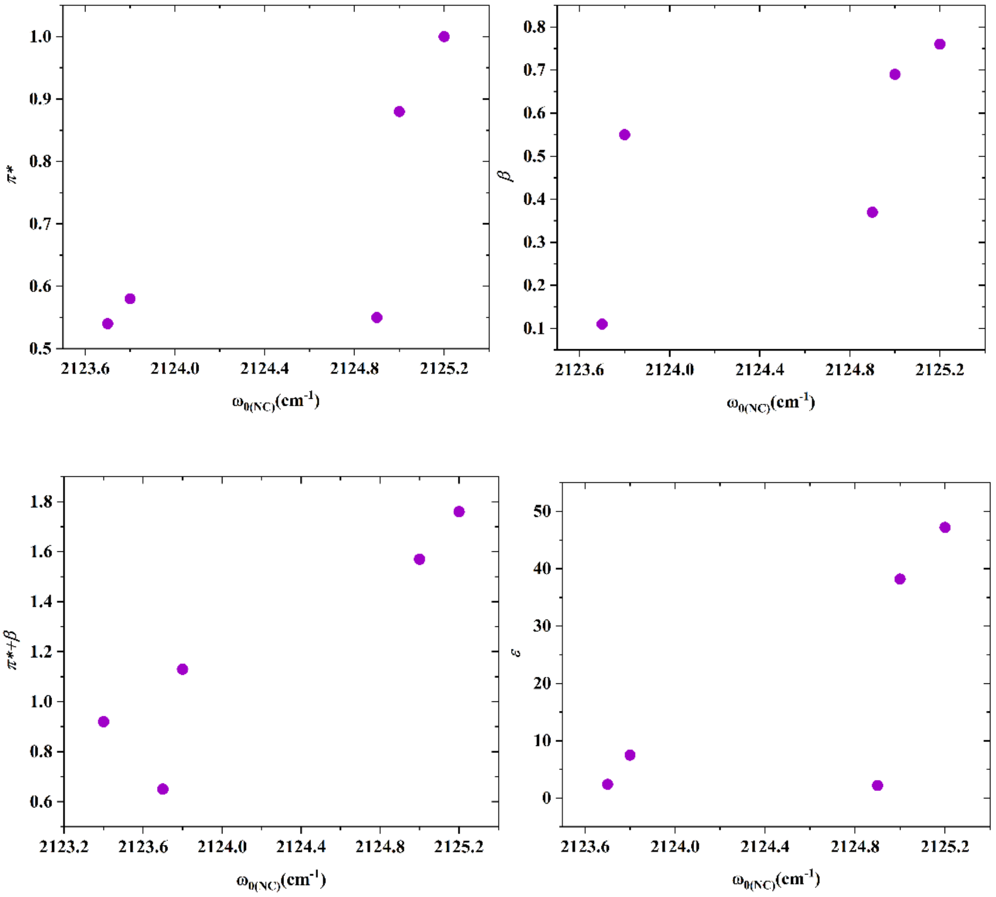

Figure S3. No linear relationships were observed between $w_{0(\mathrm{NC})}$ of 6ICMI and any solvent parameters in aprotic solvents.

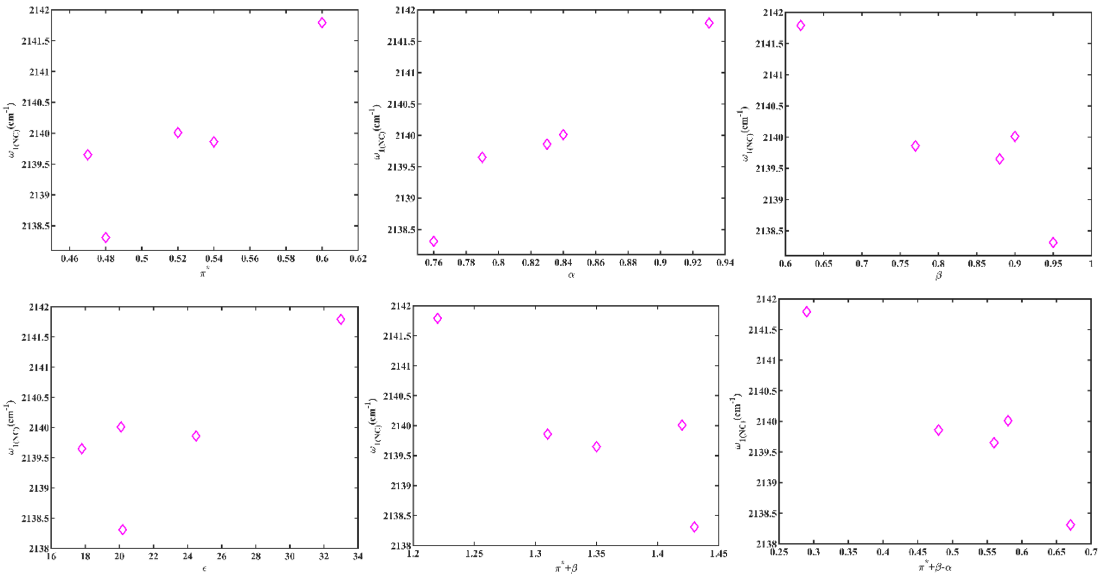

Figure S4. No linear relationships were observed between $w_{1(NC)}$ of 6ICMI and any other Kamlet-Taft parameter in studied solvents.

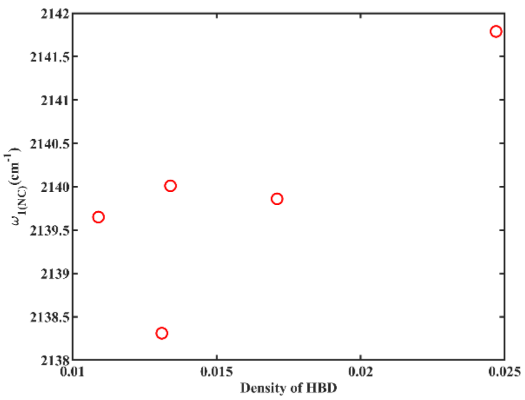

Figure S5. No linear relationships were observed between $w_{1(NC)}$ of 6ICMI and the density of hydrogen-bond donor groups in solvents.

Table S1. Frequency Parameters and solvent parameters for 5ICMI in different solvents. The center frequency ($w_{0(NC)}$, cm^{-1}), and the shoulder frequency ($w_{1(NC)}$) of the isonitrile stretching band of 5ICMI in various solvents and each solvent with its Kamlet–Taft parameters, π*(polarizability), β(hydrogen bond acceptor), α(hydrogen bond donor) and ε(dielectric constant) list in the table. KBM solvent parameter *f* is list in the table as well.

**Solvent**	$w_{0(NC)}$	$w_{1(NC)}$	**π***	**β**	**α**	**ε**	***f***
n-Octanol	2120.0	2139.2	0.4	0.81	0.77	10.3	0.43
THF	2120.8		0.58	0.55	0	7.5	0.41
n-butanol	2120.9	2139.6	0.47	0.88	0.79	17.8	0.46
Toluene	2121.0		0.54	0.11	0	2.4	0.24
2-propanol	2121.1	2136.7	0.48	0.95	0.76	20.2	0.46
n-propanol	2121.2	2140.0	0.52	0.9	0.84	20.1	0.46
Ethanol	2121.4	2139.8	0.54	0.77	0.83	24.5	0.47
DMF	2121.8		0.88	0.69	0	38.2	0.48
DMSO	2122.0		1	0.76	0	47.2	0.48
1,4-Dioxane	2122.1		0.55	0.37	0	2.2	0.22
CCl_4	2122.2		0.28	0	0	2.2	0.22
MeOH	2122.4	2141.8	0.6	0.62	0.93	33	0.48
Acetonitrile	2125.1		0.75	0.31	0.19	37.5	0.48
DCM	2125.2		0.82	0.1	0.13	8.9	0.42

Table S2. Frequency Parameters and solvent parameters for 2NI in different solvents. The center frequency ($w_{0(NC)}$, cm^{-1}) of the isonitrile stretching band of 2NI in various solvents and each solvent with its Kamlet–Taft parameters, π*(polarizability), β(hydrogen bond acceptor), α(hydrogen bond donor) and ε(dielectric constant) list in the table.

**Solvent**	$w_{0(NC)}$	π^*	**β**	**α**	**ε**
n-butanol	2122.7	0.47	0.88	0.79	17.8
2-propanol	2123.3	0.48	0.95	0.76	20.2
n-propanol	2123.2	0.52	0.90	0.84	20.1
Ethanol	2123.7	0.54	0.77	0.83	24.5
MeOH	2124.9	0.6	0.62	0.93	33
DCM	2126.9	0.82	0.1	0.13	8.9
Acetonitrile	2127.8	0.75	0.31	0.19	37.5
